# Paranasal Sinus Squamous Cell Carcinoma and Adenocarcinoma: A SEER Database Analysis

**DOI:** 10.1002/cam4.71703

**Published:** 2026-03-24

**Authors:** Lacy Brame, Spencer Hall, Aniruddha Parikh, Avigeet Gupta, Daniel Zhao, Kibwei McKinney, Lurdes Queimado

**Affiliations:** ^1^ Department of Otolaryngology Head and Neck Surgery Icahn School of Medicine at Mount Sinai New York New York USA; ^2^ Department of Biostatistics & Epidemiology University of Oklahoma Health Sciences Center Oklahoma Oklahoma USA; ^3^ Department of Otolaryngology Head and Neck Surgery The University of Oklahoma Health Sciences Center Oklahoma USA; ^4^ SSM Health Medical Group, Otolaryngology – Head and Neck Surgery Oklahoma Oklahoma USA; ^5^ Department of Cell Biology The University of Oklahoma Health Sciences Center Oklahoma USA; ^6^ TSET Health Promotion Research Center, Stephenson Cancer Center, the University of Oklahoma Health Sciences Center Oklahoma USA

**Keywords:** adenocarcinoma, paranasal sinus tumors, squamous cell carcinoma, survival analysis

## Abstract

**Background:**

Paranasal sinus tumors are rare malignancies that are known to be aggressive with poor outcomes. Data are limited regarding factors associated with survival and prognosis. In this study, we investigated factors associated with survival for both patients with squamous cell carcinoma (SCC) and adenocarcinoma (AC).

**Methods:**

The Surveillance, Epidemiology and End Results (SEER) data was utilized from the years 2000 to 2019. Kaplan–Meier survival analysis and Cox regression modeling were employed to evaluate the relationship between several co‐variates and overall survival (OS) and disease‐specific survival (DSS) among patients with SCC and AC.

**Results:**

A total of 5276 patients with SCC and 5222 patients with AC were included. Compared to SCC patients, those with AC were younger and presented with less differentiated and more advanced tumors (*p* < 0.0001). The median OS for SCC was 203 months compared to the 56 months in patients with AC (*p* < 0.0001). Primary site, race, stage, grade, and treatment modalities utilized were significantly predictive of OS and DSS in SCC patients in univariate analysis. Among AC patients, we found stage, grade, and treatment modalities to be significantly predictive of OS and DSS in univariate analysis. For SCC, stage (*p* < 0.001), primary site (*p* < 0.001), and treatment (*p* < 0.001) were significant predictors of survival on multivariate analysis. Specifically, nasal SCC was found to have improved survival compared to other sites. For AC, stage (*p* < 0.001) and treatment (*p* < 0.001) were significant predictors of survival on multivariate analysis. For both SCC and AC, treatment with both radiation and surgery had improved survival compared to radiation alone (*p* < 0.0001).

**Conclusion:**

SCC and AC of the paranasal sinus and nasal cavity portend an overall poor prognosis with limited survival. Our study effectively elucidates factors associated with survival which may be useful in treatment and counseling patients with paranasal sinus AC and SCC.

## 1 Introduction

Malignancies of the nasal cavity and paranasal sinuses are rare and comprise only around 0.2% of total cancers and only about 3%–5% of tumors of the head and neck [1]. Moreover, the incidence of these tumors has been reported to be 0.556/100,000 cases per year with a male to female ratio of 2:1 [1]. Squamous cell carcinoma (SCC) and adenocarcinoma (AC) are the most common histologic subtypes and account for 60%–70% of all sinonasal malignancies [2].

SCC and AC have been described as having an intermediate prognosis. In comparison, esthesioneuroblastoma has a relatively good prognosis whereas mucosal melanoma and sinonasal undifferentiated carcinoma (SNUC) portend a poor prognosis [3]. The most common location for these tumors is the nasal cavity followed by the maxillary sinus, ethmoid sinus, sphenoid sinus and frontal sinus, respectively [3]. Risk factors for sinonasal malignancies have been reported to include exposure to wood or leather dust, nickel, Epstein–Barr virus, salted fish, and high‐risk strains of human papilloma virus [4, 5, 6]. Numerous studies have also found an association between first and second hand smoke and the development of both sinonasal disease and malignancy [7, 8]. Patients often present once the tumor has grown large enough to affect surrounding structures. Symptoms include nasal obstruction, mucopurulent drainage, rhinorrhea, epistaxis, facial pain and pressure, and epiphora [9]. Sinonasal tumors have the capability to cause significant morbidity, especially if invasion of surrounding structures including the orbits, cranial nerves, or intracranial cavity occur. The mainstay of treatment includes surgical resection and radiation therapy with possible chemotherapy in the neoadjuvant or adjuvant setting. Radiation can also be employed in early stage tumors or in combination with surgical resection [10]. Studies are limited in regard to elucidating which factors are associated with poor survival among individuals with SCC and AC, which is likely due to how rare these tumors are in the population. However, from previous studies involving SEER data up to 2012, increased age was noted to be a negative predictor of both SCC and AC survival, while increased stage was a negative predictor only for SCC and increased grade was a negative predictor only for AC [2]. The objective of our study was to use the SEER database to evaluate survival among those with SCC and AC of the paranasal sinuses and nasal cavity. Additionally, we sought to identify factors predictive of overall survival for both SCC and AC.

## 2 Materials

Data was obtained from the National Cancer Institute (NCI) Surveillance, Epidemiology, and End Results (SEER) registry from 2000 to 2019. Permission was granted from the NCI SEER program for use and dissemination of findings from this database. The study employed deidentified population‐based data and was exempt from Institutional Review Board approval. Individuals with SCC and AC were included for analysis. The histological codes included 8050–8089 for SCC and 8140–8389 for AC. Site data were standardized using the International Classification of Disease for Oncology. Primary sites were restricted to the nasal cavity [C30.0], maxillary sinus [C31.0], ethmoid sinus [C31.1], frontal sinus [C31.2] and sphenoid sinus [C31.3]. Additional variables were collected including age, race, stage, grade, partnership status, primary site, sex, radiation status, surgery status, treatment type, and vital status. Any data which did not fit these criteria were excluded from the study. Additionally, American Joint Committee on Cancer (AJCC) staging was unable to be obtained to due differences in staging classifications over time.

### 2.1 Statistical Analysis

The Kaplan–Meier method was used to estimate the distributions of overall and disease‐specific survival for SCC and AC. Survival curves were generated using the log‐rank test. Cox proportional hazard regressions were built to model the hazard for SCC and AC based on demographics and treatment information found in the SEER database. Statistical tests were performed at significance level α = 0.05. Only lower 95% confidence interval bounds are given for groupings where too few progression events occurred during follow‐up for a full 95% confidence interval to be constructed. Stepwise selection and likelihood ratio testing were used to determine which covariates to include in the final model. All analyses were conducted using SAS version 9.4 software.

## 3 Results

**FIGURE 1:**
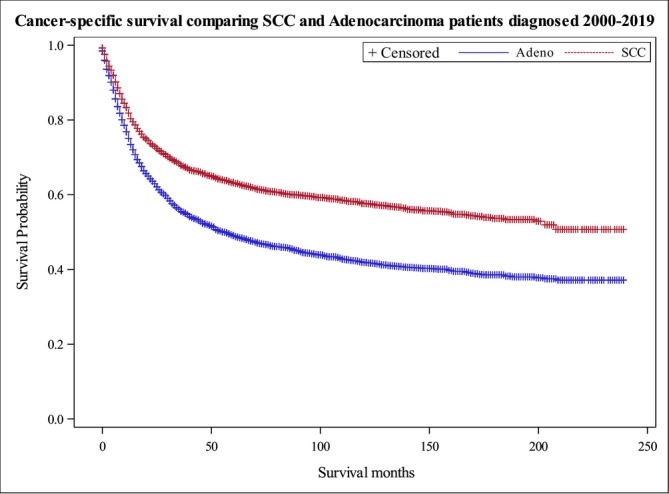
Kaplan–Meier Curves Depicting DSS between SCC and AC (*p* < 0.0001).

**FIGURE 2:**
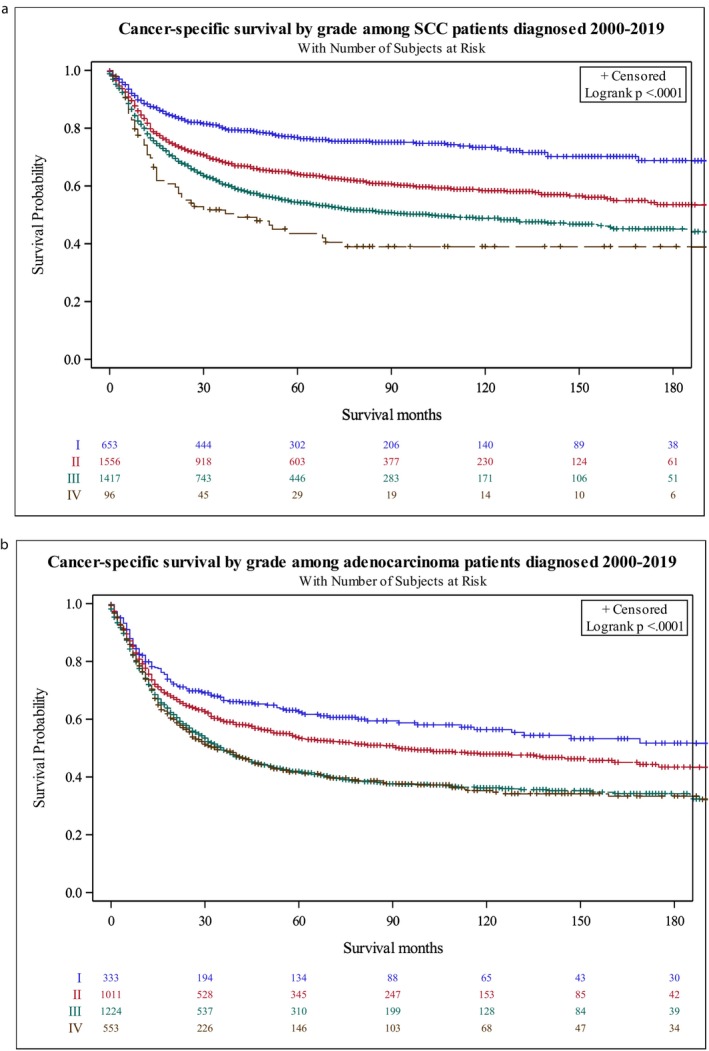
(a) Kaplan–Meier Curves depicting the effect of tumor grade on DSS for SCC. (*p* < 0.0001). (b) Kaplan–Meier Curves depicting the effect of tumor grade on DSS for AC. (*p* < 0.0001).

A total of 10,498 patients from the SEER registry were obtained. Baseline characteristics for these patients are displayed in Table 1. Data was stratified by SCC versus AC, age, race, stage, grade, partnership status, primary site, sex, radiation status, surgery status, treatment type, and vital status. Overall, 5276 individuals with SCC and 5222 with AC were included in the study. Compared to SCC patients, AC patients were significantly younger (*p* < 0.001), had a lower male/female ratio (*p* < 0.001), and were more frequently seen in non‐white individuals. Of critical clinical relevance, AC was about two times more frequent than SCC among individuals 49 and younger, suggesting more aggressive screening and treatment might be needed. Additionally, AC occurred most frequently at the maxillary sinus (62% versus 37%) while SSC was most common in the nasal cavity (53% versus 19%) (*p* < 0.001). Overall, AC patients presented more frequently with higher grades (*p* < 0.001) and advanced stages (*p* < 0.0001), and were more frequently treated with surgery plus radiation (*p* < 0.0001) (Table 1). The median cancer specific survival for SCC was found to be 203 months compared to the 56 months in patients with AC (*p* < 0.0001) (Figure 1). Kaplan–Meier curves documented that the survival of patients with SCC and AC differed significantly by grade and site of tumor (Figure 2). Univariate and multivariate analysis were performed for both SCC and AC to explore the relationships between the different covariates and survival outcomes (Tables 2, 3, 4, 5).

**TABLE 1:** Baseline characteristics for squamous cell carcinoma and adenocarcinoma patients.

		Overall (*n* = 10,498)	Diagnosis		*p*
			Adenocarcinoma (*n* = 5222)	SCC (*n* = 5276)	
Age	49 and younger	1774 (16.90%)	1109 (21.24%)	665 (12.60%)	< 0.0001
	50–64	3398 (32.37%)	1624 (31.10%)	1774 (33.62%)	
	65+	5326 (50.73%)	2489 (47.66%)	2837 (53.77%)	
Race	White	8339 (79.43%)	4010 (76.79%)	4329 (82.05%)	< 0.0001
	Black	1095 (10.43%)	614 (11.76%)	481 (9.12%)	
	Other	1064 (10.14%)	598 (11.45%)	466 (8.83%)	
Stage	Localized	2381 (23%)	615 (12%)	1766 (33%)	< 0.0001
	Regional	6555 (62%)	3797 (73%)	2758 (52%)	
	Distant	1562 (15%)	810 (16%)	752 (14%)	
Grade	Well differentiated; Grade I	986 (9%)	333 (11%)	653 (18%)	< 0.0001
	Moderately differentiated; Grade II	2567 (24%)	1011 (32%)	1556 (42%)	
	Poorly differentiated; Grade III	2641 (25%)	1224 (39%)	1417 (38%)	
	Undifferentiated; anaplastic; Grade IV	649 (6%)	553 (18%)	96 (3%)	
Partnership	Single	4391 (42%)	2217 (42%)	2174 (41%)	0.0001
	Partnered	5509 (52%)	2758 (53%)	2751 (52%)	
	Unknown	598 (6%)	247 (5%)	351 (7%)	
Primary site	Nasal	3445 (33%)	638 (19%)	2807 (53%)	< 0.0001
	Maxillary	5185 (49%)	3215 (62%)	1970 (37%)	
	Sphenoid	468 (4%)	347 (7%)	121 (2%)	
	Ethmoid	1202 (11%)	895 (17%)	307 (6%)	
	Frontal	198 (2%)	127 (2%)	71 (1%)	
Sex	Female	3856 (37%)	2069 (40%)	1787 (34%)	< 0.0001
	Male	6642 (63%)	3153 (60%)	3489 (66%)	
Radiation	No radiation treatment	3856 (36.73%)	1733 (33.19%)	2123 (40.24%)	< 0.0001
	Had radiation treatment	6642 (63.27%)	3489 (66.81%)	3153 (59.76%)	
Surgery	No surgery	3501 (33.35%)	1894 (36.27%)	1607 (30.46%)	< 0.0001
	Had surgery	6997 (66.65%)	3328 (63.73%)	3669 (69.54%)	
Treatment	Both surgery and radiation	4312 (41%)	2291 (44%)	2021 (38%)	< 0.0001
	Neither	1171 (11%)	696 (13%)	475 (9%)	
	Radiation only	2330 (22%)	1198 (23%)	1132 (21%)	
	Surgery only	2685 (26%)	1037 (20%)	1648 (31%)	
Vital status	Alive	3921 (37%)	1828 (35%)	2093 (40%)	< 0.0001

**TABLE 2:** Univariate SCC disease specific survival.

Variable	HR (95% CI)	*p*
Sex		
Male	1	
Female	0.996 (0.938–1.057)	0.8895
Age group		
< 50	1	
50–64	0.951 (0.867–1.042)	0.2813
65+	0.950 (0.870–1.037)	0.2479
Stage*		
Localized	1	
Regional	1.352 (1.270–1.438)	**< 0.0001**
Distant	1.436 (1.311–1.574)	**< 0.0001**
Race*		
White	1	
Other	1.091 (0.986–1.207)	0.0913
Black	1.149 (1.040–1.268)	**0.0063**
Partnership status*		
Single	1	
Partnered	0.954 (0.899–1.011)	0.1138
Cancer site*		
Maxillary	1	
Nasal	0.736 (0.693–0.783)	**< 0.0001**
Ethmoid	0.994 (0.8777–1.127)	0.9266
Sphenoid	0.940 (0.770–1.146)	0.5392
Frontal	0.918 (0.715–1.178)	0.5002
Surgery*		
Surgery + radiation	1	
Surgery only	0.869 (0.814–0.927)	**< 0.0001**
Radiation*		
Radiation + surgery	1	
Radiation only	1.175 (1.093–1.264)	**< 0.0001**
Grade*		
I	1	
II	1.112 (1.011–1.223)	**0.0286**
III	1.212 (1.100–1.335)	**< 0.0001**
IV	1.384 (1.109–1.728)	**0.0040**

*Note:* The bold represents significance at a *p* < 0.05.

* Indicates covariates included for consideration in multivariate analysis.

### 3.1 Squamous Cell Carcinoma

**FIGURE 3:**
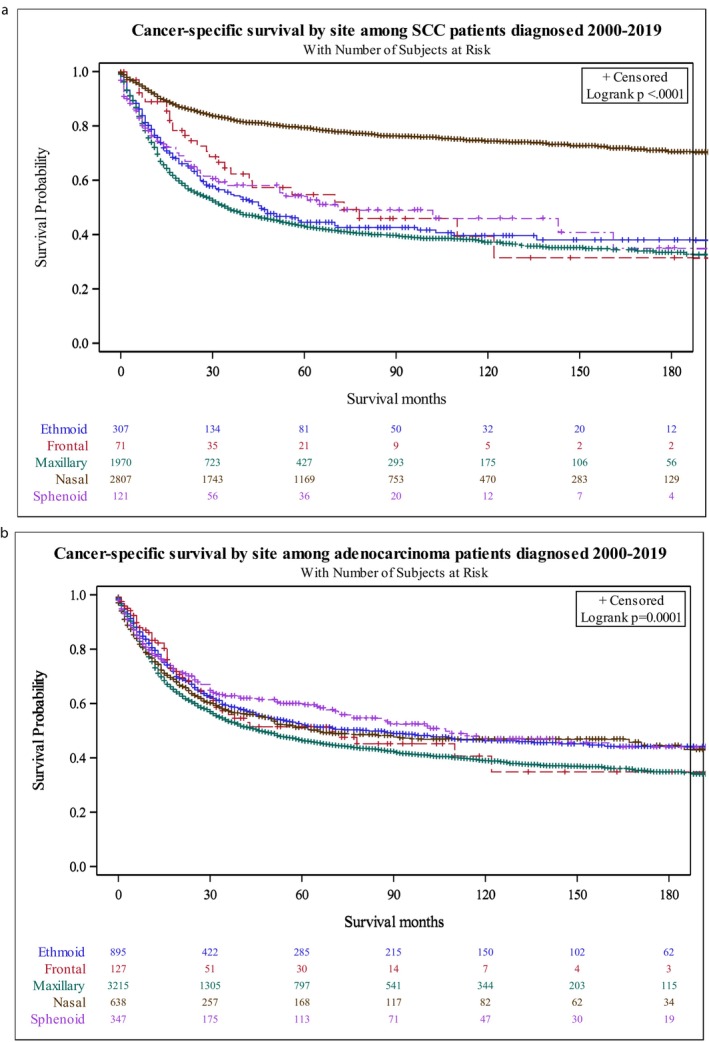
(a) Kaplan–Meier Curve depicting effect of primary site on DSS for SCC (*p* < 0.0001). (b) Kaplan–Meier Curve depicting effect of primary site on DSS for AC (*p* < 0.0001).

Univariate analysis was conducted to explore the relationship between sex, age, race, partnership status, cancer site, grade, stage, surgery, and radiation (Table 2). We found that stage was significantly predictive of disease specific survival (*p* < 0.0001). Specifically, we found a worse survival with distant disease (HR = 1.436) and regional disease (HR = 1.352) compared to localized disease. Our results also demonstrated differences in survival by race (*p* = 0.0063). We found that black individuals had worse survival than white individuals (HR = 1.149). Interestingly, we also found differing survival by cancer site. Nasal cavity cancer was associated with improved survival (Figure 3 and Table 3). Grade was also significantly predictive of survival with worse survival seen for a higher grade (Table 2 and Figure 2b). Surgery alone compared to both surgery and radiation was associated with improved survival (HR = 0.869, Table 2). Moreover, radiation alone compared to both surgery and radiation was associated with worse survival (HR = 1.175, Table 2). Upon stepwise selection for multivariate testing, three variables were retained in the final model: stage, site, and treatment modality (Table 3). After adjusting for stage and treatment type, those who were diagnosed with nasal SCC as the primary site of cancer had a hazard ratio of 0.789 (95% CI: 0.734–0.847) compared to those that were diagnosed with maxillary SCC (Table 3; *p* < 0.0001). Moreover, cancer staging that was classified as “regional” or “distant” was associated with greater hazard (HR = 1.162 and 1.340, respectively) for SCC compared to “localized” cancers (*p* < 0.001). From this model, we can see radiation alone was associated with greater disease‐specific hazard (HR: 1.182; 95% CI: 1.099–1.272) for SCC than radiation plus surgery (Table 3). Interestingly, race and grade were significant in univariate analysis (Table 2) but they did not remain significant after controlling for other variables.

**TABLE 3:** Multivariate SCC disease specific survival.

Variable	HR (95% CI)	*p*
Stage		
Localized	1	
Regional	1.162 (1.075–1.257)	**0.0002**
Distant	1.340 (1.217–1.476)	**< 0.0001**
Cancer site		
Maxillary	1	
Nasal	0.789 (0.734–0.847)	**< 0.0001**
Ethmoid	0.981 (0.865–1.112)	0.7605
Sphenoid	0.888 (0.728–1.084)	0.2440
Frontal	0.926 (0.722–1.189)	0.5476
Surgery		
Surgery + radiation	1	
Surgery only	0.992 (0.924–1.066)	0.8348
Radiation		
Radiation + surgery	1	
Radiation only	1.182 (1.099–1.272)	**< 0.0001**

*Note:* The bold represents significance at a *p* < 0.05.

### 3.2 Adenocarcinoma

Univariate analysis was conducted to explore the relationship between sex, age, race, partnership status, cancer site, stage, grade, surgery, and radiation (Table 4). Similar to SCC, we found that stage (Table 4 and Figure 3) was significantly predictive of survival for AC (*p* < 0.0001). Specifically, we found a worse survival with distant disease (HR = 1.420) and regional disease (HR = 1.256) compared to localized disease (*p* < 0.0001). For AC, there were no observable differences in survival on univariate analysis for age, race, partnership status, cancer site, or surgery (Table 4). In univariate analysis, grade was significantly predictive of survival, with worse survival seen with a higher grade (Table 4 and Figure 3b). However, this association was not seen in multivariate analysis. We did see a difference in survival based on radiation therapy. Interestingly, individuals that received radiation alone had a worse survival than those that received radiation with surgery (HR = 1.194, Table 4). Upon stepwise selection for multivariate testing, two variables were retained in the final model: treatment modality and stage (Table 5). This analysis confirmed that distant (HR = 1.350; 95% CI 1.199–1.519) and regional (HR = 1.220; 95% CI: 1.111–1.340) staging had a higher hazard ratio compared to localized staging. After adjusting for stage at diagnosis, those who were diagnosed and treated with radiation alone for AC had a hazard ratio of 1.178 (95% CI: 1.098–1.264) compared to those who were treated with both surgery and radiation. Our multivariate modeling demonstrates that surgery was important for minimizing hazard of adenocarcinoma after controlling for stage at diagnosis. Overall, our multivariate analysis demonstrated that treatment type and stage were important for predicting survival in this patient population.

**TABLE 4:** Univariate AC disease specific survival.

Variable	HR (95% CI)	*p*
Sex		
Male	1	
Female	0.971 (0.915–1.031)	0.3401
Age group		
< 50	1	
50–64	1.042 (0.961–1.129)	0.3200
65+	1.050 (0.974–1.132)	0.2040
Stage*		
Localized	1	
Regional	1.256 (1.147–1.375)	**< 0.0001**
Distant	1.420 (1.265–1.593)	**< 0.0001**
Race		
White	1	
Other	0.964 (0.879–1.058)	0.4437
Black	1.056 (0.964–1.157)	0.2396
Partnership status		
Single	1	
Partnered	0.963 (0.907–1.023)	0.2257
Cancer site		
Maxillary	1	
Nasal	0.924 (0.843–1.013)	0.0939
Ethmoid	0.966 (0.893–1.045)	0.3850
Sphenoid	0.933 (0.828–1.051)	0.2538
Frontal	0.953 (0.788–1.152)	0.6159
Surgery*		
Surgery + radiation	1	
Surgery only	0.933 (0.867–1.004)	0.0655
Radiation		
Radiation + surgery	1	
Radiation only	1.194 (1.113–1.281)	**< 0.0001**
Grade		
I	1	
II	1.068 (0.937–1.216)	0.3252
III	1.191 (1.048–1.354)	**0.0073**
IV	1.175 (1.018–1.357)	**0.0278**

*Note:* The bold represents significance at a *p* < 0.05.

* Indicates covariates included for consideration in multivariate analysis.

**TABLE 5:** Multivariate AC disease specific survival.

Variable	HR (95% CI)	*p*
Stage*		
Localized	1	
Regional	1.220 (1.111–1.340)	**< 0.0001**
Distant	1.350 (1.199–1.519)	**< 0.0001**
Surgery*		
Surgery + radiation	1	
Surgery only	0.972 (0.901–1.048)	0.4628
Radiation		
Radiation + surgery	1	
Radiation only	1.178 (1.098–1.264)	**< 0.0001**

*Note:* The bold represents significance at a *p* < 0.05.

## 4 Discussion

Our study utilized data from the SEER database, which is extensive and generalizable to the United States population. Data was obtained from the 2000 to 2019 registry which is the update to previous papers and displays current trends in both survival and risk factors related to poor prognosis in this patient population. Among 10,498 patients, pathology was found to be a key factor associated with survival. Specifically, we found that SCC patients had a greater disease specific survival compared to AC which is consistent with other studies describing survival of these tumor types (Figure 1) [11]. Curiously, a previous SEER database study reporting 3714 cases between 1973 and 2012 documented a better survival for AC than for SCC [2]. Specifically, that study documented a 5‐year cancer specific survival of 57.5% for AC and 39.1% for SCC. We observed a median cancer specific survival for AC of 56 months, which is similar to the data reported previously. Our observed 10‐year survival for AC is also similar to the previously reported. These data suggest that the prognosis for AC has not changed significantly in the last decade. However, we observed a median cancer specific survival for SCC of 203 months; this is much higher than the previously reported 10‐year disease specific survival of 33.5% for SCC [2]. These data reflect in part the large portion of nasal SCC included in our series but also suggest that the prognosis for SCC has improved dramatically in the past decade.

For SCC, on univariate analysis factors associated with worse survival included race, cancer site, stage, grade, and treatment modality. Although not significant, we found that partnered individuals had better survival when compared to single individuals (Table 2). Moreover, we found that African Americans had lower survival when compared to Caucasians and other races (Table 2). For SCC, surgery was also associated with increased odds of survival when compared to those who did not undergo surgery (Table 2). Surgery has been shown to increase survival in SCC patients in previous studies [2]. For AC, factors associated with significantly worse survival included stage, radiation only therapy, and grade (Table 4). Similar to SCC, although not significant, we found improved survival in partnered individuals compared to unpartnered individuals.

Prior studies evaluating other disease processes have demonstrated a link between marital status and health outcomes [12, 13]. The literature reports that unmarried individuals have poorer health and have an overall higher mortality risk compared to married individuals [12]. Unmarried patients also reportedly present with more advanced stage, have less access to care, and have lower socioeconomic status compared to married individuals, which likely results in poorer disease survival [14]. Additionally, some authors propose that this improved survival among married individuals may be due to these individuals reporting less stress, engaging in less risky health behaviors, and having more social support overall compared to unmarried individuals [13]. Moreover, partnered individuals reported positive health practices including avoidance of heavy smoking or drinking and participation in healthy behaviors such as exercise and good dietary habits [15]. Additionally, evidence has suggested that partnered men are more likely to attend routine health maintenance examinations compared to their single counterparts, a trend often attributed to spousal influence prompting seeking out a healthcare appointment [16]. We hypothesize that this may allow for earlier detection of malignancy in partnered individuals. Our study was unable to obtain information on alcohol consumption or tobacco utilization. This may be an interesting future avenue for research with primary data.

Our study found that survival varied by race, with African‐Americans having a lower survival for squamous cell carcinoma (Table 2). This is consistent with previous literature demonstrating variable survival across race [17, 18]. Racial and cultural differences in the United States are complex, with disparities present among races in access to care, quality of care provided, and health outcomes. Prior studies have found that poverty, lack of health insurance, poor health behaviors, inadequate education, unsafe living environments, and limited financial means are the underpinnings resulting in health disparities in the United States [19]. In particular, poor access to care affects a patient's opportunity to obtain a timely diagnosis and adequate treatment, which is of utmost importance for malignant lesions.

On multivariate analysis, only tumor site, advanced stage, and treatment modality were predictive of survival. A stepwise Cox regression was used to identify the influence of predictors on overall and disease‐specific survival of both SCC and AC. At each step, variables were added based on an entry threshold, and the stepwise function evaluated at each step if they belonged in the final model. Despite our very large series, tumor grade was not significantly associated with cancer‐specific survival. This observation differs from previous reports and might reflect the stringency of our methodology.

We observed that for SCC, nasal cavity cancers have the best prognosis and highest survival, which is unsurprising. The nasal cavity is a confined space, so even small tumors can narrow the airway, causing nasal obstruction which may lead to earlier patient presentation. Additionally, patients may present earlier due to symptoms such as epistaxis or nasal discharge, which are more noticeable given the accessible location of this site. Further insight might have been gained by assessing whether SCC arising from inverted papilloma contributed to this observation. Unfortunately, this variable is not available within the SEER database. Intriguingly, cancer site did not significantly impact survival in AC patients, suggesting that these tumors behave very differently than SCC even when originating in the nasal cavity.

For both SCC and AC, we found poorer survival with more advanced stage. This is unsurprising and may be due to these cancers being either locally unresectable, resected with positive margins, or having metastatic disease present at time of diagnosis. This may be improved in the future due to advances in craniofacial resection and reconstructive techniques and improvements in chemoradiation therapy. For example, tumors that have occupied the anterior cranial fossa and orbit were deemed as “unresectable” in the past, but surgical advances have improved approaches to allow for resection of these tumors with less morbidity [20]. Additionally, recent advances in neo‐adjuvant chemotherapy options may allow for improved survival in previously unresectable tumors [21].

Our multivariate analysis demonstrated that, for both SCC and AC, patients who received combined surgery and radiation had improved survival compared to those treated with radiation alone. This difference may reflect the fact that larger or more advanced tumors are often only amenable to radiation. Primary radiation therapy is typically reserved for patients with inoperable tumors or those who are not surgical candidates [22]. Of note radiation is a dichotomous variable in the SEER database, thus we could not access the impact of proton beam therapy versus general radiation therapy or the dosage of radiation which was administered.

A strength of our study is the inclusion of a multivariate analysis, which was built using stepwise selection of significant univariate variables. The findings from this analysis merit particular emphasis given the rigorous variable selection process. A limitation to the SEER database is the lack of information regarding surgical margins, orbit involvement, and intracranial involvement. Future studies are needed to evaluate survival among individuals with extensive spread both into the orbital region and intracranially, which have been shown to be associated with poor survival in previous studies [23]. We were also constrained by the limited availability of morbidity variables. Additionally, AJCC staging data varied significantly over time for sinonasal malignancies, thus the extracted AJCC staging data were not suitable for comparison across time. Additionally, SEER data do not include a variable for systemic therapy. These limitations have been similarly noted in other SEER‐based studies on sinonasal malignancies [2].

An additional limitation of our study is the stringency of our analysis which restricted a number of variables that were included in the multivariate analysis due to the stepwise selection process. Future studies are warranted to assess the impact of the excluded variables on survival outcomes using multivariate modeling.

In summary, our study demonstrates several factors predictive of survival in both SCC and AC patients. Understanding factors associated with poorer survival is critical for clinicians in educating patients, providing care, and improving clinical outcomes. Efforts must be made to curb disparities and improve access to care, both from a clinical and public health perspective.

## 5 Conclusion

Both SCC and AC are rare tumors of the nasal cavity and paranasal sinuses with a high rate of mortality and morbidity. For SCC patients, our study demonstrated differences in survival by race, primary site, stage, grade, and treatment modalities utilized. For AC patients, our study demonstrated differences in survival by stage, grade, and treatment modalities utilized. Prior studies have implicated that social determinants of health, health disparities, and inequities all influence the development and progression of disease in United States populations [18, 24]. Understanding factors associated with poor survival from these tumors will aid in guiding treatment and understanding disparities that may exist among this patient population.

## Author Contributions

Lacy Brame: Conceptualization, methodology, investigation, data curation, writing original draft, writing review and editing. Spencer Hall: Methodology, data curation, formal analysis, writing original draft. Aniruddha Parikh: Conceptualization, methodology, investigation, writing original draft, writing review and editing. Avigeet Gupta: Data curation, writing review and editing. Daniel Zhao: Methodology, data curation, formal analysis, writing review and editing, supervision. Kibwei McKinney: Conceptualization, methodology, writing review and editing, supervision. Lurdes Queimado: Conceptualization, methodology, writing review and editing, supervision.

## Funding

This work was supported in part by the National Cancer Institute (NCI) of the National Institutes of Health (RO1CA242168, P30CA225520) and the Oklahoma Tobacco Settlement Endowment Trust Health Promotion Research Center. Dr. Queimado holds a Presbyterian Health Foundation Endowed Chair in Otorhinolaryngology Position. The funders had no role in study design, data collection, and analysis, decision to publish, or preparation of the manuscript.

## Disclosure

Kibwei McKinney is a member of the Medtronic and Sanofi speaker‘s bureau. There are no other disclosures for the remainder of the authors.

## Conflicts of Interest

The authors declare no conflicts of interest.

## Data Availability

The data that support the findings of this study are available from the corresponding author upon reasonable request.
